# Prognostic significance of CT-determined sarcopenia in older patients with advanced squamous cell lung cancer treated with programmed death-1 inhibitors

**DOI:** 10.1038/s41598-024-62825-2

**Published:** 2024-05-26

**Authors:** Lin Ying, Liqian Xu, Ji Yang, Qin Zhang

**Affiliations:** 1 Department of Geriatrics, School of Medicine, The First Affiliated Hospital, Zhejiang University, Hangzhou, 310003 Zhejiang People’s Republic of China; 2 Zhejiang Provincial Key Laboratory for Diagnosis and Treatment of Aging and Physic-Chemical Injury Diseases, School of Medicine, The First Affiliated Hospital, Zhejiang University, Hangzhou, 310003 Zhejiang People’s Republic of China

**Keywords:** Sarcopenia, Squamous cell lung cancer, Programmed death-1 inhibitors, Prognosis, Cancer, Medical research

## Abstract

Sarcopenia has been associated with higher toxicity induced by anti-cancer treatments and shorter survival in patients with squamous cell lung carcinoma (SqCLC). Over the past few decades, immune checkpoint inhibitors (ICIs) significantly improves the prognosis. However, few clinical studies explored the effectiveness of immunotherapy in the elderly population. Here, we performed a retrospective analysis to determine the prognostic role of sarcopenia in older patients with SqCLC receiving ICIs. We retrospectively assessed SqCLC patients who were treated with PD-1 inhibitors and all patients were at least 70 years old. Pre-treatment sarcopenic status was determined by analyzing L3 skeletal muscle index (SMI) with chest CT. Progression-free survival (PFS), disease-specific survival (DSS) and overall survival (OS) were estimated using the Kaplan–Meier method, and the differences in survival were compared using the log-rank test. Among 130 male SqCLC patients, 93 had sarcopenia. Patients with sarcopenia were older and had a lower body mass index (BMI). Over an average follow-up of 20.8 months, 92 patients died. For all 130 patients, the mean OS was 13.3 months. Patients with sarcopenia had a significantly shorter OS and PFS than those without sarcopenia (OS, 12.4 ± 5.2 months vs. 15.5 ± 10.5 months, P = 0.028; PFS, 6.4 ± 2.9 months vs. 7.7 ± 4.2 months; P = 0.035). Multivariable analysis showed that sarcopenia was an independent prognostic factor for shorter OS and PFS. CT-determined sarcopenia is an independent prognostic factor for older patients with SqCLC receiving ICIs.

## Introduction

In recent years, there has been an upward trend of lung cancer incidence and mortality in China. Lung cancer is still the major reason for cancer-related deaths^1,2^. Non-small cell lung cancer (NSCLC) accounts for approximately 80% of primary lung cancers. Most NSCLCs are adenocarcinomas or squamous cell lung carcinomas (SqCLCs), with SqCLCs accounting for approximately 35% of NSCLCs^3,4^.

Over the past few decades, targeted therapy has greatly improved the survival time and quality of life of patients with lung adenocarcinoma with targetable driver gene mutations^5,6^. However, the benefits of targeted therapy are limited in SqCLC because of the limited understanding of molecular targets^7^. Rather than single oncogenic driver mutations, SqCLC is often driven by the overexpression or amplification of multiple receptors^8^. As a result, only a few targeted drugs are available for SqCLC. Fortunately, immune checkpoint inhibitors (ICIs), such as anti-programmed cell death protein 1 (PD-1) or anti-programmed cell death ligand 1 (PD-L1) monoclonal antibodies, have become an option for patients with advanced SqCLC, in addition to radiotherapy, chemotherapy, and targeted therapy. Immunotherapy significantly improves the prognosis of patients with SqCLC ^9^.

Sarcopenia, defined as low muscle strength and muscle mass^10^, is prevalent in patients with primary tumors, and its incidence in patients with lung cancer is as high as 61%^11^. Sarcopenia is related to systemic chronic inflammation, mitochondrial dysfunction, and imbalance of synthesis and metabolism^12^. The clinical importance of sarcopenia in cancer patients has attracted increasing interest over the past decade. However, the pathogenesis is still not completely clear. It is noteworthy that patients may be sarcopenic even if their nutritional status is maintained or they are obese. Patients with sarcopenia exhibit higher chemotherapeutic toxicity and poorer compliance to cancer treatment. A wide body of literature has shown that the prognosis of patients with NSCLC and sarcopenia is worse than that of NSCLC patients without sarcopenia^13–15^. However, the effect of sarcopenia in patients with SqCLC undergoing ICI treatment remains unclear.

With global aging, the proportion of older adults with SqCLC is increasing. Older adults often have a variety of comorbidities and a decline in organ reserve function, which lead to a decline in the tolerance to chemotherapy. Previous studies have shown that the incidence of neutropenia and infection after chemotherapy in patients aged > 65 years are significantly higher than those in younger patients. In addition, the risk and severity of the adverse effects of chemotherapy also increase with age^16,17^. A major difficulty in the treatment of older patients with advanced SqCLC is that most clinical trials have strict criteria for the admission and discharge of older adults, making these studies not representative of older patients. Finding the most appropriate treatment for older patients with cancer is challenging for clinicians. Here, we performed a retrospective analysis to determine the prognostic role of sarcopenia in older patients with SqCLC receiving PD-1 inhibitors.

## Methods

### Patients

This study was approved by the Ethics Committee of the First Affiliated Hospital, College of Medicine, Zhejiang University (IRB: IIT20170524B).We retrospectively identified 138 patients with advanced (stage IIIB–IV) or recurrent SqCLC who were treated with PD-1 inhibitors (pembrolizumab, camrelizumab, sintilimab, nivolumab, tislelizumab, or toripalimab) between November 2017 and January 2021 at our institution, with the final follow-up occurring on January 31, 2022. All patients were at least 70 years of age and received 200 mg of PD-1inhibitors intravenously every 3 weeks. The clinical and auxiliary examination data were integrated. Written informed consent was obtained from all subjects.

The key exclusion criteria included history of autoimmune disease, primary or secondary immunodeficiency, chronic active viral hepatitis, or uncontrolled central nervous system metastasis. Because SqCLC has a male predominance, we also excluded women and included only men.

### Definition of sarcopenia

Dual-energy X-ray absorptiometry is the preferred go-to method for the measurement of skeletal muscle mass because of accuracy and repeatability^18^. However, this technology is not widely used in all departments of our hospital; therefore, pre-treatment (within one month) computed tomography (CT) was used to evaluate body composition to determine sarcopenia. CT scans were performed on a 320-row detector CT scanner (120 kVp tube voltage, automatic tube current), and reconstruction thickness was 1.25 mm. Two observers read and analyzed the CT images. We used the third lumbar vertebra (L3) skeletal muscle mass index (SMI) as an estimator of sarcopenia. The cross-sectional area of the skeletal muscle at the L3 level was measured using ImageJ software (National Institutes of Health, Bethesda, MD, USA). The skeletal muscle area included the psoas, erector spinae, quadratus lumborum, transversus abdominis, external and internal oblique, and rectus abdominis muscles. The CT HU thresholds were − 29 to + 150 for quantifying muscle^19^. The SMI was calculated as follows: SMI (cm^2^/m^2^) = cross-sectional area (cm^2^)/height^2^ (m^2^). Sarcopenia was defined as an SMI < 52.4 cm^2^/m^2^ for men, according to previous studies^15,20^.

### Data collection

Baseline demographics, comorbidities (the age-adjusted Charlson comorbidity index score, A-CCI), eastern cooperative oncology group performance status (ECOG PS), previous treatment history, laboratory results, and PD-L1 positivity were collected. The ACCI was calculated by adding the comorbidity score and the age (Table 1)^21,22^. PD-L1 expression in the tumor samples was assessed by immunohistochemistry (IHC) using a PD-L1 IHC kit (22C3; Dako, Denmark) and characterized according to the tumor proportion score (TPS).

**Table 1:** Scoring rules of age-adjusted charlson comorbidity index.

Score	Comorbidity
(1) Comorbidity score	
1	Uncomplicated diabetes mellitus, mild liver disease, congestive heart failure, myocardial infarction, chronic obstructive pulmonary disease, dementia, connective tissue disease, peptic ulcer disease, peripheral vascular disease, cerebrovascular accident or transient ischemic attack
2	Diabetes mellitus, moderate to severe chronic kidney disease, end-organ damage diabetes mellitus, localized solid tumor, leukemia, lymphoma
3	Moderate to severe liver disease
6	Metastatic solid tumor, acquired immune deficiency syndrome

Score	Age (years)
(2) Age score	
3	70–79
4	≥ 80

Response evaluation was performed using a CT scan of the target lesions and classified according to the immune-related response evaluation criteria in solid tumors^23^. Evaluations were performed every 8 weeks. The co-primary endpoints were PFS (the time from initial treatment to clinical or radiographic progression or death), DSS (the time from initial treatment to death due to cancer) and OS (the time from initial treatment to death due to any cause). Secondary endpoints included the objective response rate (ORR, the proportion of patients whose best overall response was complete or partial response [CR, PR]) and the disease control rate (DCR, the proportion of patients whose best overall response was CR, PR, or stable disease [SD]).

We also evaluated the safety. Patients were monitored for adverse events (AEs) from the first dose of study treatment to 90 days after the last dose. AEs were graded using the National Cancer Institute common terminology criteria for adverse events (version 5.0).

### Statistics

Quantitative data were presented as mean ± standard deviation. The Kolmogorov–Smirnov test was performed to test for normality. Quantitative variables were compared using student’s *t*-test for independent samples or the non-parametric Mann–Whitney test. Differences between categorical variables were compared using the chi-square test. Survival curves were constructed using the Kaplan–Meier method, and differences in survival were calculated using the log-rank test. Cox proportional hazards regression analysis was used to identify predictors of PFS and OS. We analyzed the following variables in multivariable analysis: sarcopenia, age, smoking history, BMI, ACCI, ECOG PS, PD-L1 TPS score, serum albumin, history of lung surgery and radiation therapy. P < 0.05 was considered to denote significance. Statistical analyses were performed using the SPSS software (version 22.0; SPSS, Chicago, IL, USA) and Prism (GraphPad, Software, La Jolla, CA, USA).

***Ethical statement.***

This study was approved by the Ethics Committee of the First Affiliated Hospital, College of Medicine, Zhejiang University (IRB:IIT20170524B), China and conducted in accordance with the principles of the Declaration of Helsinki.

## Results

### Patient demographics and baseline characteristics

Between November 2017 and January 2021, 138 patients with SqCLC who received anti-PD-1 antibodies were eligible. The 130 male patients were included. The baseline characteristics of the 130 study participants are summarized in Table 2. They were all over 70 years old, and the mean age was 74.9 ± 3.5 years (range, 70–86 years). All patients were pathologically confirmed and had stage 3 or 4 disease.

**Table 2:** Baseline patients characteristics.

Characteristics	All (n = 130)	Sarcopenia (n = 93)	No sarcopenia (n = 37)	P value
Age (years), mean ± SD	74.9 ± 3.5	75.4 ± 3.8	73.5 ± 2.0	0.004
Smoking status, n (%)				
Never smoker	34 (26.2%)	23 (24.7%)	11 (29.7%)	0.650
Past smoker	39 (30%)	30 (32.3%)	9 (24.3%)	
Current smoker	57 (43.8%)	40 (43%)	17 (46%)	
BMI (kg/m^2^), mean ± SD	20.7 ± 1.5	20.4 ± 1.4	21.5 ± 1.4	< 0.001
BMI, n (%)				
Underweight (< 18.5 kg/m^2^)	14 (10.8%)	12 (12.9%)	2 (5.4%)	0.237
Normal (18.5–22.9 kg/m^2^)	104 (80%)	74 (79.6%)	30 (81.1%)	
Overwei%ght (23.0–24.9 kg/m^2^)	11 (8.5%)	7 (7.5%)	4 (10.8%)	
Obesity (≥ 25.0 kg/m^2^)	1 (0.7%)	0	1 (2.7%)	
ACCI, n (%)				
3–4	41 (31.5%)	30 (32.3%)	11 (29.7%)	0.158
5–6	55 (42.3%)	35 (37.6%)	20 (54.1%)	
≥ 7	34 (26.2%)	28 (30.1%)	6 (16.2%)	
ECOG PS , n (%)^a^				
0	26 (20%)	10 (10.8%)	16 (43.2%)	< 0.001
1	51 (39.2%)	39 (41.9%)	12 (32.4%)	
2–3	53 (40.8%)	44 (47.3%)	9 (24.3%)	
Serum albumin, mean ± SD	37.2 ± 4.0	36.7 ± 4.1	38.5 ± 3.4	0.012
CRP, median (IQR)	13.3 (5.9, 26.4)	13.2 (6.9, 30.5)	13.5 (2.9, 19.0)	0.030
IL-6, median (IQR)	15.9 (10.0, 25.8)	16.1 (10.2, 27.5)	15.8 (9.0, 22.2)	0.047
L3 SMI (cm^2^/m^2^), mean ± SD	48.9 ± 6.7	45.7 ± 4.5	57.1 ± 3.6	< 0.001
PD-L1 expression, n (%)				
≥ 50	32 (24.6%)	24 (25.8%)	8 (21.6%)	0.687
1–49	62 (47.7%)	46 (49.5%)	16 (43.2%)	
< 1	31 (23.8%)	20 (21.5%)	11 (29.7%)	
Indeterminate	5 (3.8%)	3 (3.2%)	2 (5.4%)	
Previous lung surgery, n(%)				
Yes	33 (25.4%)	24 (25.8%)	9 (24.3%)	0.861
No	97 (74.6%)	69 (74.2%)	28 (75.7%)	
Previous radiation therapy, n (%)				
Yes	15 (11.5%)	12 (12.9%)	3 (8.1%)	0.440
No	115 (88.5%)	81 (87.1%)	34 (91.9% )	
First-line chemotherapy, n (%)				
Combination therapy	89 (83.2%)	59 (80.8%)	30 (88.2%)	0.340
Single-agent	18 (16.8%)	14 (19.2%)	4 (11.8%)	
Chemotherapy regimen, n (%)				
Paclitaxel/platinum	81 (75.7%)	54 (74.0%)	27 (79.4%)	0.616
Gemcitabine/platinum	8 (7.5%)	5 (6.8%)	3 (8.8%)	
Paclitaxel	18 (16.8%)	14 (19.2%)	4 (11.8%)	
Receipt of second-line therapy, n				
EGFR TKIs	4	3	1	0.809
Docetaxel	3	2	1	

*SD* standard deviation, *BMI* body mass index, *ACCI* the age-adjusted Charlson comorbidity index, *CRP* C-reactive protein, *IQR* interquartile range, *IL-6* interleukin 6, *SMI* skeletal muscle mass index, *PD-L1* programmed cell death protein 1 ligand 1, *EGFR* epidermal growth factor receptor, *TKI* tyrosine kinase inhibitor.

^a^Eastern Cooperative Oncology Group performance status (ECOG PS) ranges from 0 to 5, with 0 indicating no symptoms and higher scores indicating greater disability.

### Prevalence of and factors associated with sarcopenia

Among the 130 patients, 93 (71.5%) had sarcopenia based on the L3 SMI cutoff. Patients with sarcopenia had a mean SMI of 45.7 cm^2^/m^2^, whereas those without sarcopenia had a mean SMI of 57.1 cm^2^/m^2^ (P < 0.001). As summarized in Table 2, patients with sarcopenia were older, had a lower body mass index (BMI), and were more frequently underweight than those without sarcopenia (12.9% vs. 5.4%). The proportion of ACCI scores ≥ 7 was significantly higher in the sarcopenia group than in the non-sarcopenic group (30.1% vs. 16.2%). However, no significant difference was found between two groups in terms of ACCI and smoking history. The majority of non-sarcopenic patients had an ECOG PS of 0–1 (75.7%), whereas 47.3% patients with sarcopenia had an ECOG PS of 2–3. Although the baseline levels of serum albumin, C-reactive protein (CRP), and interleukin 6 (IL-6) were comparable between patients with and without sarcopenia, the development of sarcopenia was significantly associated with lower albumin levels and higher CRP and IL-6 levels.

Previous treatment histories, such as surgery and radiotherapy, did not differ according to sarcopenic status; among the 130 patients, 103 received first-line chemotherapy, mainly paclitaxel or gemcitabine combined with platinum or paclitaxel alone. Seven patients received second-line treatment for general intolerance or other reasons, such as targeted drugs and docetaxel. There was no significant difference between the two groups in terms of the choice of chemotherapy regimen.

### Prognostic significance of sarcopenia in patients with SqCLC

**Figure 1:**
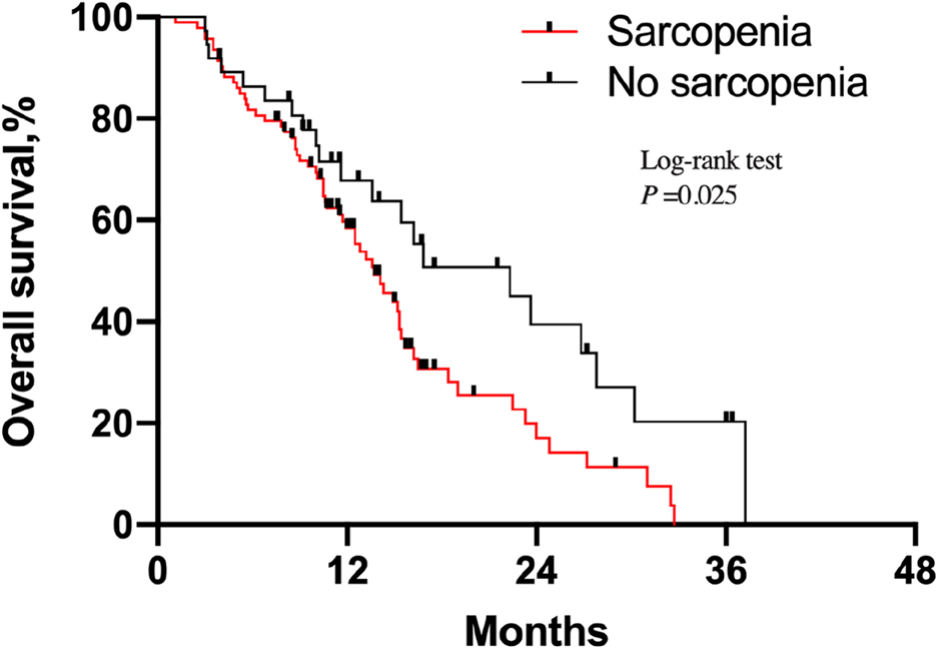
Kaplan–Meier estimates of overall survival in SqCLC patients treated by programmed death-1 (PD-1) inhibitors according to sarcopenic status.

**Figure 2:**
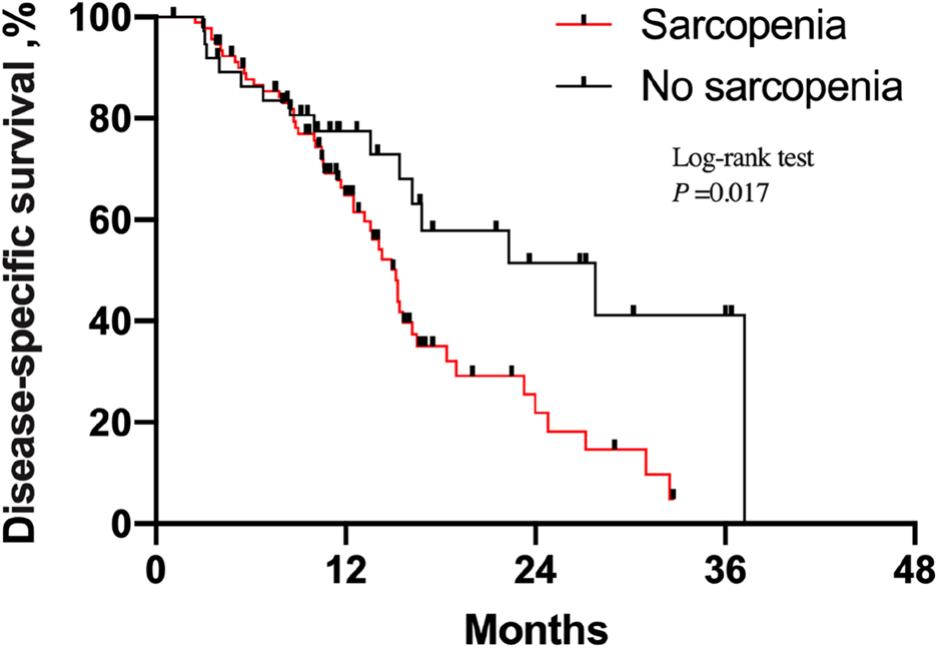
Kaplan–Meier estimates of disease-specific survival in SqCLC patients treated by programmed death-1 (PD-1) inhibitors according to sarcopenic status.

**Figure 3:**
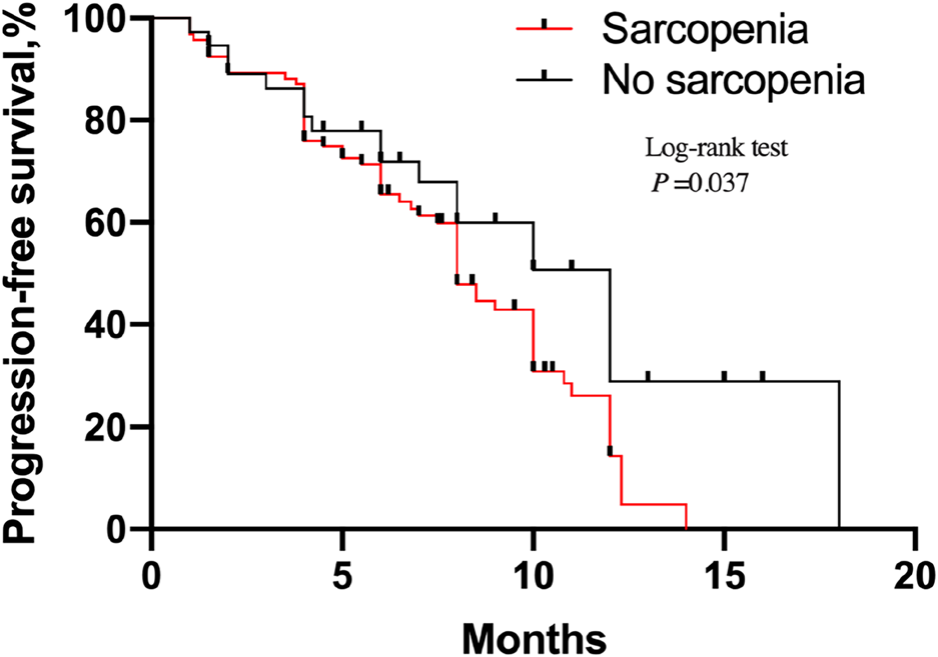
Kaplan–Meier estimates of progression-free survival in SqCLC patients treated by programmed death-1 (PD-1) inhibitors according to sarcopenic status.

Of the 93 patients with sarcopenia, two (2.2%) patients achieved CR, eight (8.6%) achieved PR, and 27 (29.0%) achieved SD, resulting in ORR and DCR values of 10.8% and 39.8%, respectively. The ORR and DCR in the non-sarcopenic group were 16.2% and 43.2%, respectively (Table 3). Over an average follow-up of 20.8 months (95% CI 18.5–23.0), 92 patients (70.8%) died. For all 130 patients, the mean OS was 13.3 months (95% CI 12.0–14.5). Patients with sarcopenia had a significantly shorter OS and PFS than those without sarcopenia (OS, 12.4 ± 5.2 months vs. 15.5 ± 10.5 months, P = 0.028; PFS, 6.4 ± 2.9 months vs. 7.7 ± 4.2 months; P = 0.035). Kaplan–Meier curves showed that sarcopenia was an independent prognostic factor for shorter OS (HR 1.69; 95% CI 1.067–2.668; P = 0.025), DSS (HR, 1.88; 95% CI 1.122–3.150; P = 0.017) and PFS (HR 1.71; 95% CI 1.034–2.826; P = 0.037) (Figs. 1, 2, 3). In addition to sarcopenia, advanced age (≥ 80 years) and poor PS were poor prognostic factors (Table 4).

**Table 3:** Response to treatment according to sarcopenic status.

Response	Sarcopenia (n = 93)	No sarcopenia (n = 37)	Total (n = 130)
Complete response	2 (2.2%)	1 (2.7%)	3 (2.3%)
Partial response	8 (8.6%)	5 (13.5%)	13 (10%)
Stable disease	27 (29.0%)	10 (27.0%)	37 (28.5%)
Progressive disease	56 (60.2%)	21 (56.8%)	77 (59.2%)

Data are represented as number of patients with percentages.

**Table 4:** Multivariate analysis of factors influencing OS and PFS.

	Overall survival			Progression-free survival		
	HR	95% CI	P	HR	95% CI	P
Sarcopenia (yes/no)	1.693	1.067–2.686	0.025	1.710	1.034–2.826	0.037
Age (≥ 80/ < 80)	2.384	1.178–4.822	0.005	2.022	1.045–3.915	0.036
Smoking history (never/current or former smoker)	0.757	0.465–1.230	0.229	0.864	0.539–1.385	0.502
BMI (< 23/ ≥ 23)	1.157	0.582–2.301	0.691	1.016	0.488–2.115	0.964
ACCI (< 7/ ≥ 7)	0.763	0.457–1.275	0.264	0.717	0.425–1.209	0.145
ECOG PS (2–3/0–1)^a^	1.714	1.093–2.688	0.019	1.910	1.212–3.010	0.001
PD-L1 TPS score (≥ 50%/ < 50%)	0.690	0.436–1.088	0.124	0.766	0.459–1.276	0.305
Serum albumin (≥ 35 g/L/ < 35 g/L)	0.762	0.484–1.200	0.240	0.827	0.517–1.321	0.426
History of lung surgery (yes/no)	0.609	0.388–0.956	0.047	0.640	0.406–1.010	0.058
History of radiation therapy (yes/no)	1.321	0.706–2.473	0.384	1.474	0.810–2.681	0.232

*PFS* progression-free survival, *OS* overall survival, *HR* hazard ratio, *CI* confidence interval, *BMI* body mass index, *ACCI* the age-adjusted Charlson comorbidity index, *PD-L1* programmed cell death protein 1 ligand 1, *TPS* tumor proportion score.

^a^Eastern cooperative oncology group performance status (ECOG PS) ranges from 0 to 5, with 0 indicating no symptoms and higher scores indicating greater disability.

### Safety analysis

At the time of this analysis, the median (range) duration of exposure with ICIs was 6.7 (0.03‒23.6) months among patients in the sarcopenia group and 7.9 (0.03‒28.1) months among patients in the non-sarcopenic group. Overall, 92 (98.9%) of the 93 patients in the sarcopenia group and 35 (94.6%) of the 37 patients in the non-sarcopenic group experienced one or more AEs (Table 5). AEs of grade 3 or higher were reported in 70 patients (75.3%) in the sarcopenia group and 24 patients (64.9%) in the non-sarcopenic group, with the most common ones being neutropenia (69 [74.2%] vs 24 [64.9%]), anemia (61 [65.6%] vs 21 [56.8%]) , alopecia (58 [62.4%] vs 24 [64.9%]) and decreased appetite (51 [54.8%] vs 18 [48.6%]). AEs leading to discontinuation of treatment occurred more frequently in the sarcopenia group (44, 47.3%) than in the non-sarcopenic group (11, 29.8%).

**Table 5:** Incidence of all-cause AEs and immune-mediated AEs.

Event	Sarcopenia (n = 93)		No sarcopenia (n = 37)	
Any AE, n (%)	92 (98.9%)		35 (94.6%)	
Grade 3–5	70 (75.3%)		24(64.9%)	
Leading to treatment discontinuation	44 (47.3%)		11(29.8%)	
Treatment-related adverse events, n (%)^a^	Any grade	Grade 3–5	Any grade	Grade 3–5
Neutropenia	69 (74.2%)	42 (45.2%)	24 (64.9%)	13 (35.1%)
Anemia	61 (65.6%)	38 (40.9%)	21 (56.8%)	10 (27.0%)
Alopecia	58 (62.4%)	3 (3.2%)	24 (64.9%)	1 (2.7%)
Decreased appetite	51 (54.8%)	12 (12.9%)	18 (48.6%)	3 (8.1%)
Nausea	39 (41.9%)	6 (6.5%)	16 (43.2%)	2 (5.4%)
Thrombocytopenia	36 (38.7%)	9 (9.7%)	14 (37.8%)	4 (10.8%)
Fatigue	34 (36.6%)	10 (10.8%)	10 (27.0%)	3 (8.1%)
Peripheral neuropathy	19 (20.4%)	4 (4.3%)	7 (18.9%)	3 (8.1%)
Vomiting	17 (18.3%)	5 (5.4%)	6 (16.2%)	3 (8.1%)
Rash	14 (15.1%)	3 (3.2%)	6 (16.2%)	1 (2.7%)
Immune-related adverse events, n (%)^b^	28 (30.1%)		12 (32.4%)	
Hypothyroidism	10 (10.8%)		4 (10.8%)	
Adrenal insufficiency	9 (9.7%)		3 (8.1%)	
Rash	7 (7.5%)		3 (8.1%)	
Hepatitis	6 (6.5%)		2 (5.4%)	
Pneumonitis	5 (5.4%)		3 (8.1%)	
Colitis	3 (3.2%)		2 (5.4%)	

*AE* adverse event.

^a^Treatment-related adverse events occurring in 15% or more of patients in either group were listed. Events are shown in descending order of frequency in the sarcopenia group.

^b^Immune–related adverse events occurring in 3% or more of patients in either group were listed.

A total of 28 of the 93 patients (30.1%) in sarcopenia group and 12 of the 37 patients (32.4%) in non-sarcopenic group had immune-related adverse events. The most common immune-related adverse event was hypothyroidism (10 [10.8%] vs 4 [10.8%]), adrenal insufficiency (9 [9.7%] vs 3 [8.1%]) and rash (7 [7.5%] vs 3 [8.1%]).

## Discussion

In this study, we demonstrated that a high SMI was independently associated with a lower risk of mortality. To the best of our knowledge, this is the largest clinical study evaluating the association between skeletal muscle mass and outcomes in older patients with SqCLC who received ICIs. Currently, there are few studies on older patients with SqCLC. To fill this gap, our study included patients aged > 70 years with advanced SqCLC. All patients received ICIs, and the mean OS was 13 months, which is similar to the survival period of SqCLC reported in other studies. In our study, the OS, DSS and PFS of patients with sarcopenia were relatively shorter; therefore, SMI could be a useful variable for predicting prognosis.

Cancer patients with sarcopenia often have a worse prognosis than cancer patients without sarcopenia. This may manifest as poor efficacy of chemotherapy or immunotherapy. Melanoma patients with sarcopenia are more likely to experience ipilimumab-related toxicity^24^, and patients with advanced NSCLC treated with nivolumab or pembrolizumab had poorer survival outcomes and response rates if they have sarcopenia^25^. Although the ways in which sarcopenia has a negative effect on the clinical efficacy of ICIs remain unclear, there are several potential explanations^26^. This could involve the interplay between the skeletal muscles and the immune system. Several animal experiments and epidemiological studies have revealed a causal relationship between chronic inflammation and tumorigenesis. Chronic inflammation also plays an important role in the development of sarcopenia, such as increased level of TGF-βand IL-6, which is a cause of muscle atrophy. These cytokines mediate T cell exhaustion, which causes immune dysregulation and resistance to ICIs^27^. Moreover, skeletal muscle, as an endocrine organ, secretes cytokines that regulate immunity and are involved in the modulation of the immune response^28^. This internal immune factor may explain the poor therapeutic effect and short survival period of patients with skeletal muscle reduction.

There are other important factors that need to be considered. Our study showed that patients with sarcopenia tended to be thin and had low plasma albumin levels. These patients have poor tolerance to chemotherapy and immunotherapy and are at a high risk of side effects, such as bone marrow suppression and other side effects after chemotherapy or immunotherapy. Although increased BMI has been shown to have protective effects on the prognosis of some patients with cancer^29,30^, some evidence suggests that obesity contributes to oncologic development and progression via chronic inflammation^31^. Therefore, BMI is not a perfect biomarker of body composition as it relies heavily on adiposity and cannot effectively represent body composition^32^.

We also discussed the impact of comorbidities on prognosis. In the study, we used ACCI to evaluate the incidence of comorbidities. It is worth noting that there is no difference in ACCI between the two groups and it cannot be used to predict prognosis. It's easy to understand that even if two patients have the same comorbidities, their physical functions can differ significantly. So ACCI cannot represent the overall situation. ECOG PS score is actually more suitable for evaluating the condition and treatment tolerance of the elderly. In our clinical work, we are also gradually carrying out comprehensive geriatric assessment (CGA)^33^. We believe that in future research, there can be a better evaluation system to comprehensively evaluate the function of elderly people.

Despite the novelty of this study, it had several limitations. First, this was a single-institution retrospective study with a small cohort. However, to the best of our knowledge, this is the largest study investigating the influence of skeletal muscle mass on clinical outcomes in older patients with SqCLC treated with PD-1 inhibitors. Second, this was a retrospective study and was subject to selection bias. We attempted to mitigate the effects of selection bias by including all patients treated at our center with available clinical data and baseline CT images. However, our patients were generally older and many had a history of smoking, which is often associated with cardiovascular and cerebrovascular diseases. We only analyzed whether ACCI can predict prognosis, but did not specifically discuss the impact of these underlying comorbidities on patient survival. Third, due to the application of multiple anti PD-1 drugs in our study, there may be differences in treatment efficacy caused by differences in the agents. Finally, we only included skeletal muscle as a surrogate for body composition and did not include other marker such as contents of fat. In future research, we can explore the impact of changes in body composition on the prognosis of patients receiving immunotherapy, not limited to muscle mass.

In conclusion, baseline sarcopenia was associated with significantly poorer outcomes after PD-1 inhibitor treatment in older patients with advanced SqCLC. In clinical practice, screening for sarcopenia can help identify patients who are likely to achieve a long-term response.

## Data Availability

All data generated or analysed in this study are included in this article.
